# Loss of Happiness for Land-Expropriated, Urbanised Residents: A Comparison Based on Multiple Groups

**DOI:** 10.3390/ijerph19042425

**Published:** 2022-02-19

**Authors:** Junhui Han, Zenghui Huo, Xuejia Sun

**Affiliations:** 1 School of Economics and Management, Taiyuan University of Technology, Taiyuan 030024, China; hanjunhui@tyut.edu.cn (J.H.); sunxuejia@tyut.edu.cn (X.S.); 2 College of Economics & Management, China Jiliang University, Hangzhou 314423, China

**Keywords:** urbanisation, rural-to-urban hukou conversion, happiness, suppressing effect

## Abstract

Are land-expropriated, urbanised residents living happily? This question is not only related to the quality of urbanisation but also has important reference for evaluating the reform of the land acquisition system. On the basis of the Chinese General Social Survey data in 2017 and 2018, the HeckProbit model, the ordered probit model and the mediating effect model were used to study the happiness and underlying mechanism of land-expropriated, urbanised residents. The results showed that the older the farmers are, the lower the probability of obtaining urban hukou through land acquisition, and women will be more likely to achieve household registration through land requisition. Education and party status have significant inhibitory effects on hukou conversion through land acquisition. A comparison among multiple groups indicated that the happiness level of each group is ranked as follows: residents who attained urban hukou through education and job recruitment > urban native residents > land-expropriated, urbanised residents > farmers; however, the happiness level of the latter two groups has no significant difference. The propensity score matching method was further used to reduce the endogeneity due to selection bias, and the results were still robust. The lower self-assessment of socioeconomic status indirectly caused the loss of happiness of land-expropriated, urbanised residents. However, the high living expectation ‘suppressed’ the negative effect of land acquisition on happiness to some extent.

## 1. Introduction

In China, the household registration system or hukou was introduced in 1958. This system categorises Chinese citizens into agricultural and nonagricultural hukou or groups. For a long time, those with urban or nonagricultural hukou have been able to access city services and facilities including hospitals, housing and eldercare. On the contrary, those with rural hukou cannot enjoy these benefits. Since the reform and opening up, China has witnessed the largest and fastest urbanisation in the world. Statistics show that China’s urbanisation rate increased from 17.92% in 1978 to 60.65% in 2020. During the 14th Five-Year Plan period, China’s urbanisation rate will continue to rise to the target level of 65%. On the one hand, China’s urbanisation has optimised the regional industrial structure; on the other hand, it has nurtured a large number of landless farmers. According to the national general land use planning (2016–2030), there will be approximately 40 million land-expropriated farmers from 2020 to 2030 (Lv et al., 2015) [1]. In addition, with the increasing land acquisition disputes in recent years, the Chinese government has launched a tentative land acquisition reform centred on acquisition procedures, compensation and resettlement and has actively deepened the comprehensive reform of the land system and the household registration system. One of its characteristics is that land-expropriated farmers can apply for nonagricultural household registration due to the construction of national or local governments. Thus, land-expropriated, urbanised residents appeared in China.

However, the conversion from agricultural to nonagricultural household registration through land requisition is a ‘passive’ choice of farmers with highly strong policy implications in most cases. Residents who enter cities passively face many risks, such as difficulties in building social networks and poor ability to participate in the market. In addition, the government failed to take measures to protect their fundamental interests. As a result, serious social problems are often caused (Cai et al.,2007; Li, 2013) [2,3]. Therefore, the livelihood of land-expropriated farmers after ‘entering the city’ has attracted considerable attention. The newly revised ‘Law of the People’s Republic of China on Land Administration’ in 2019 clearly stipulates that the living standards of land-expropriated farmers cannot be lowered, and their long-term livelihoods shall be guaranteed. Happiness is an important index to evaluate the results of urbanisation (Huo et al., 2018) [4]. Thus, who realises the conversion of household registration through land acquisition? For this group, how are they doing? Are they happier? Exploring such questions will not only help improve the quality of China’s urbanisation but can also provide important reference for the evaluation of the reform of the household registration system.

## 2. Literature Review

### 2.1. Factors Influencing Farmers’ Willingness to Have Their Land Expropriated

Xu et al. (2011) claimed that the age of farmers has a negative effect on their willingness for land acquisition [5]. That is, as the age increases, the working ability of farmers drops. If they lose their land, then getting a job again will be difficult for them; thus, they are unwilling to give up their land. Mu et al. (2009) indicated that the older the farmers are, the deeper their affection for the land and the lower their willingness for land acquisition [6]. However, Xu et al. (2018) found that the older the farmers are, the higher their willingness for land acquisition [7]. The research conducted by Luo et al. (2011) showed that education has a positive effect on farmers’ willingness for land acquisition [8]. The reason is that the higher the education level is, the more likely they will be to master certain nonagricultural employment skills; hence, they favour land acquisition. However, farmers with higher education may be ‘prejudiced’ against the land acquisition system, which will lead to uncertainty in the relationship between education and the willingness for land acquisition. In addition, the family demographic structure will also affect the willingness for land acquisition. Wang et al. (2013) found that families with a high dependency ratio rely heavily on land. Personal or family income also affects their willingness for land acquisition [9]. Wang et al. (2009) reported that the higher the proportion of nonagricultural income of farmers before land acquisition, the more willing they will be to have their land expropriated [10]. In fact, farmers have a psychological process of comparing the income from farming with future nonagricultural income. Many scholars, such as Luo et al. (2011), have indicated that the expected living standards and employment opportunities after land acquisition will have a significant effect on the willingness of farmers to have their land expropriated [8].

### 2.2. Happiness of Land-Expropriated Farmers

The pursuit of happiness is the eternal theme of the development of human society. At the macro level, Guriev et al. (2009) and Oswald et al. (2015) believed that economic development, unemployment, inflation rate and social income inequality all affect residents’ happiness [11,12]. From a micro level, Alesina et al. (2004) reported that factors such as income, gender, age, education, health and marriage can all affect individual happiness [13]. Few studies have been conducted on the happiness of land-expropriated farmers. Many scholars, such as Chen et al. (2007), have indicated that the benefits of land-expropriated farmers are seriously damaged due to many reasons such as few employment opportunities, lack of social security system and unreasonable compensation for land requisition [14]. On the basis of the survey data in Wuhan in 2010, Yuan et al. (2012) found that the agricultural income of land-expropriated farmers is declining, whereas the cost of living is rising; moreover, the elderly have poor economic welfare [15]. With the continuous improvement of the land acquisition system and the implementation of multiple security mechanisms, the welfare of land-expropriated farmers has been improved. For example, by constructing an evaluation index system, Hu et al. (2019) discovered that the welfare of the land-expropriated farmers in Hunan Province has increased, although regional differences exist [16]. Furthermore, Jiang et al. (2021) indicated that the overall level of the sense of gain, happiness and security of land-expropriated farmers is low, which needs to be further improved [17].

One of the manifestations of the multiple security mechanisms for the land acquisition system is that land-expropriated farmers can apply for urban hukou status. As Lu (2008) mentioned, China’s household registration system not only performs the functions of population registration and management but is also closely related to social welfare [18]. Therefore, a few scholars have conducted research on land-expropriated, urbanised residents and their happiness. Lu et al. (2014) found that in comparison with urban native residents, the ‘new urban residents’ with rural-to-urban hukou conversion have higher happiness [19]. Conversely, Knight et al. (2010), Jiang et al. (2012) and Guo et al. (2018) revealed that the happiness of urban immigrants is lower than that of local residents [20,21,22]. In fact, a certain degree of heterogeneity exists among residents with rural-to-urban hukou conversion. Different pathways of conversion may have different effects on happiness. Zheng et al. (2013) divided the rural-to-urban conversion into two types: selective mobility and policy program [23]. The former refers to the conversion from rural to urban by individual ability, such as education, job recruitment and joining the army, whereas the latter refers to relying on national policies, such as land acquisition and resettlement. On this basis, Huo et al. (2018) found that the group who attained household registration through education and job recruitment has a higher happiness than the land-expropriated, urbanised residents; moreover, no significant difference was observed in the level of happiness between the land-expropriated, urbanised residents and farmers [4]. Zhao et al. (2020) also reported a similar conclusion in the sense of economic gain [24]. The reason may be that although land-expropriated, urbanised residents have obtained household registration, the education, employment and social security benefits are still absent.

### 2.3. Summary of Literature

To sum up, the heterogeneity of ‘rural-to-urban hukou conversion’ has been noted in the literature. Selective mobility and policy programs may have different effects on happiness. However, few studies have analysed the pathway differences between the two types of hukou conversion. The rural-to-urban hukou conversion through land requisition has strong policy implications. Then, who are obtaining the household registration through land acquisition? In fact, if only land-expropriated farmers are used as the research samples, then the relevant information of farmers without land acquisition will be lost, which will cause the selection bias of the sample. Combined with the characteristics of the data used and the ideas of Guo et al. (2013) [25], the rural-to-urban hukou conversion through land acquisition is decomposed into two independent but interrelated decision making. The first step is to decide whether to transfer hukou from rural to urban status. The second step is the urbanised pathway choice, that is, by land acquisition or through education and job. Combined with the above analysis, this study used the HeckProbit model for research.

In addition, a few scholars have conducted comparative studies on the happiness of land-expropriated, urbanised residents; however, the comparative objects are mostly limited to farmers without land expropriation or urban native residents. Therefore, a diversified group must be constructed through more detailed data to fully understand whether land-expropriated, urbanised residents have gained happiness. In this study, the samples were divided into four groups: farmers, urban native residents, the residents who attained urban hukou through education and job recruitment, and the land-expropriated, urbanised residents, and a comparative study was made.

In view of the above considerations, the following questions were mainly explored in this study. First, who are obtaining household registration through land acquisition? Second, in comparison with other groups, are land-expropriated, urbanised residents happier? Third, what are the mechanisms that affect the happiness of land-expropriated, urbanised residents?

## 3. Data and Model

### 3.1. Data Sources

The Chinese General Social Survey (CGSS) data in 2017 and 2018 used in this study can be applied from the China National Survey Database and the official website of the CGSS. By screening samples, Sample A had 9274 farmers; Sample B had 6704 urban native residents, Sample C had 614 land-expropriated, urbanised residents; and Sample D had 1513 residents who attained urban hukou through education and job recruitment.

### 3.2. Variable Selection

There were two types of dependent variables. The first type was the dependent variable involved in the HeckProbit model, that is, whether farmers choose the rural-to-urban hukou conversion and the conversion pathway selection. On the basis of the channels for obtaining nonagricultural household registration and Sample A in the questionnaire, the dummy variable was established to determine whether the residents have experienced the hukou conversion in the selection equation. A value of 1 was assigned to the samples who have undergone the hukou conversion; otherwise, it was 0. Then, the dummy variables of conversion pathway or mode were established in the outcome equation. If the hukou conversion was achieved through land acquisition, then the variable was assigned a value of 1; if it was achieved through education and job recruitment, then the value of the variable would be 0. Notably, in the CGSS questionnaire, there existed other hukou conversion channels in addition to land acquisition. However, this study believes that education and job recruitment are the hukou conversion mode that best reflect individual abilities and efforts and have a higher degree of self-selection. To form a sharp contrast with the group through land acquisition, only the samples who obtained urban hukou through education and job recruitment were retained here. In fact, other hukou conversion channels had a relatively small proportion. Therefore, these samples were deleted in this study. The second type was personal subjective happiness. A self-reported method was adopted to measure happiness, and ‘very unhappy’, ‘relatively unhappy’, ‘not happy or unhappy’, ‘relatively happy’ and ‘very happy’ were assigned a value of 1–5, respectively. The higher the value was, the stronger the sense of happiness.

The independent variable in this study was household registration, and different binary dummy variables were set in different regression equations. For information on using the variable, see the contents below. The control variables in the HeckProbit model mainly included age, gender, education, party status, parents’ education and parents’ party status.

The control variables in the model of happiness included age, gender, marital cohabitation, education, health status, family economic status and the number of real estates. In addition, living expectation and self-assessment of socioeconomic status were regarded as the mediating variables in the mediating model. The descriptive statistics of each variable are shown in Table 1.

### 3.3. Measurement Model

The first is the HeckProbit model. It is assumed that $Y_{i1}$, $Y_{i2}$ are dummy variables of conversion from agricultural to nonagricultural status and conversion from agricultural to nonagricultural status through land acquisition, respectively. $Y_{i1}{}^{*}$, $Y_{i2}{}^{*}$ represent the corresponding unobservable latent variables. The econometric model is set as follows:

(1) $$Y_{i1}{}^{*}=aX_{i1}+\varepsilon_{i}$$

(2) $$Y_{i2}{}^{*}=bX_{i2}+\mu_{i}$$

$\varepsilon_{i}$ and $\mu_{i}$ represent the stochastic component, and the standard normal distribution is assumed. corr(ε,μ)=rho, $X_{i1}$, and $X_{i2}$ represent the characteristic variable of the *i*th sample in the selection equation and the result equation, respectively. In order for the model to be estimated, $X_{i2}\neq X_{i1}$ needs to be satisfied (Christopher, 2006) [26] When $Y_{i1}{}^{*}$, $Y_{i2}{}^{*}$ are positive, the values of $Y_{i1}$, $Y_{i2}$ are 1; otherwise, they are 0. $Y_{i2}$ can be observed if and only if $Y_{i1}=1$. The specific formula is expressed as follows:

(3) $$Y_{i2}=\{\begin{array}{c} Y_{i2}{}^{*}\;if\;\;Y_{i1}{}^{*}>0\;\\ .\;\;\;\;\;\;\;if\;\;Y_{i1}{}^{*}\leq 0 \end{array}$$

(4) $$Y_{i1}=\{\;\begin{array}{c} 1\;if\;\;Y_{i1}{}^{*}>0\;\\ 0\;\;\;if\;\;Y_{i1}{}^{*}\leq 0 \end{array}$$

The second is the subjective happiness decision model. For ordinal-categorical variables, the following ordered probit model for happiness was built. Assuming that the value range of happiness $Y_{i}$ is 1, 2,…, m, the ordered probit model can be expressed as:

When

(5) $$u_{j-1}<Y_{i}{}^{*}\leq u_{j},j=1,2,\ldots ,m,\;Y_{i}=j$$

where $Y_{i}^{*}$ is the latent variable of the ordinal categorical variable happiness $Y_{i}$ and is affected by the control variable $X_{i}$. It can be expressed as:

(6) $$Y_{i}{}^{*}=\gamma X_{i}+u_{i}$$

Further, when $u_{j}\leq u_{j+1},u_{0}=-\infty ,u_{m}=+\infty$, the probability of $Y_{i}=j$ can be expressed by the following form:

(7) $$pr\left(Y_{i}=j\right)=\Phi \left(u_{j}-\gamma X_{i}\right)-\Phi \left(u_{j-1}-\gamma X_{i}\right)$$

where Φ represents the cumulative density function that obeys the standard normal distribution and satisfies j=1,…,5.

In addition, the mediating model and propensity score matching method were used in this study. The former mainly examines the mediating effect of the living expectation and self-assessment of socioeconomic status on the relationship between the conversion from agricultural to nonagricultural status through land acquisition and happiness; the latter was used to test the net happiness effect brought by the experience of the conversion from agricultural to nonagricultural status through land acquisition. Due to space limitations, the relevant formula derivations are no longer listed here. All models were operated by Stata15.0

## 4. Empirical Results

### 4.1. HeckProbit Model of Pathways to Hukou Conversion

In this section, the paper used the append command in Stata15.0 to merge Sample A, Sample B and Sample C. Meanwhile, dummy variables in the Outcome and Selection equation were set. The HeckProbit model was used to estimate the selection and outcome equations. The results are shown in Table 2.

As shown in Table 2, rho = 0.369 (*p* < 0.05), a sample selection bias was possible, indicating that the HeckProbit model should be used. From the selection equation, age had a positive effect on the choice of hukou conversion and was significant at the level of 0.01. In comparison with men, women had a higher probability of choosing the hukou transition, which is similar to the conclusion of Guo et al. (2018) [25]. In addition, education and party status for individuals and their parents had a positive and significant effect on the choice of the hukou transition.

From the outcome equation, the older the residents was, the less willing they would be to choose the hukou transition through land acquisition, which is consistent with the conclusions in most studies. At the same time, gender differences existed in the hukou transition through land acquisition. Women preferred to obtain household registration conversion through land acquisition, but the regression coefficient was not significant. The regression coefficient of individual education was negative and significant at the 0.01 level. Thus, the higher the level of education was, the lower the probability of choosing the hukou transition through land acquisition. Similarly, party status had a significant inhibitory effect on the choice.

### 4.2. Comparison of Happiness among Multiple Groups

In this section, the happiness was divided into five orderly levels 1–5, and multiple variables at the individual and family levels were controlled at the same time. Three ordered Probit regression models were used to compare the difference in happiness among the four groups. The household registration variable was a binary dummy variable. The paper used the append command in Stata15.0 to merge Sample A and sample C in Model 1, and the other two models underwent similar processing. In Model 1, household registration = 0 represented the farmers (Sample A), whereas household registration = 1 represented the land-expropriated, urbanised residents (Sample C). In Model 2, household registration = 0 referred to urban native residents (Sample B), whereas household registration = 1 referred to Sample C. In Model 3, household registration = 0 referred to Sample B, whereas household registration = 1 referred to the residents who attained urban hukou through education and job recruitment (Sample D). The results are displayed in Models 1–3 in Table 3.

In the three models, variables such as age, gender and marital cohabitation affected happiness in different directions and degrees. A significant positive correlation existed between age and happiness. In comparison with women, men had a lower level of happiness and was significant at the 0.01 level. The regression coefficients of marital cohabitation were positive in the three models and significant at the 0.01 level; thus, cohabitation could increase the level of happiness. In fact, living together in a couple or with a spouse could provide financial or emotional support for each other, which would increase happiness. In addition, the higher the education was, the higher the level of happiness. Health status was also an important factor affecting happiness, which is consistent with the results of most studies. Family economic status and the number of real estates had a positive effect on individual happiness.

In Model 1, the results indicated that in comparison with farmers (Sample A), the land-expropriated, urbanised residents (Sample C) had a higher level of happiness (about 0.0112 Probit units), although not significant. Thus, the hukou conversion through land acquisition did not significantly improve the happiness of these residents. This finding is similar to the research conclusion obtained by Huo et al. (2018) [4]. The control group in Model 2 was urban native residents (Sample B). After controlling for other variables, in comparison with Sample B, the happiness of the group in (Sample C) decreased slightly and was significant at the level of 0.1. In Model 3, urban native residents (Sample B) were taken as the control group, and the results showed that the happiness of the group in (Sample D) was 0.0232 Probit units higher than that of the group in (Sample B) and was significant at the level of 0.05. By combining the three models and through comparison, the happiness level of each group was ranked as follows: residents who attained urban hukou through education and job recruitment (Sample D) > urban native residents (Sample B) > land-expropriated, urbanised residents (Sample C) > farmers (Sample A). However, no significant difference was found in the level of happiness between the latter two groups.

### 4.3. Endogeneity Test of Happiness of the Land-Expropriated, Urbanised Residents

To examine the net effect of the household registration conversion through land acquisition on happiness while minimising endogeneity due to the selection bias of the sample, the propensity score matching method was used for the test with Model 1 as an example. Specifically, the radius matching method was used to test the balance of the two groups of samples: the farmers (Sample A) and the land-expropriated, urbanised residents (Sample C). The test results are shown in Table 4.

As shown in Table 4, significant differences existed in the variables between the two groups of samples before matching. After the two groups of samples were matched, the deviation ratio of all variables fell to less than 15%. In addition, the reduction in the deviation of other variables exceeded 60%, except for gender and health status. After matching, the T value, which indicates the difference between the two groups of samples, became significantly smaller. The samples after using propensity score matching basically passed the balance test.

Table 5 shows the average treatment effect (ATT) of the treatment group, the average treatment effect (ATU) of the control group and the overall average treatment effect (ATE) estimated by the radius matching method. For the population sample, the results of ATE showed that the net effect of the happiness of the group with hukou conversion through land acquisition was about 3.21%. The ATT estimate showed that the effect of hukou conversion through land acquisition on the happiness of the treatment group was about 3.35%. The ATU value indicated the following counterfactual reasoning: if the farmers (Sample A) obtained urban hukou through land acquisition, then their happiness would be increased by 3.2%. Notably, none of the above conclusions are significant; thus, no significant difference existed in the happiness between the land-expropriated, urbanised residents (Sample C) and the farmers (Sample A). This result is consistent with the conclusion of Model 1 in Table 3. The propensity score matching method was also used to test Samples B, C and D. Conclusions similar to those of Models 2 and 3 in Table 3 were obtained. Due to space limitations, detailed results were not reported. In other words, after eliminating endogeneity due to sample selection by propensity score matching, the conclusions of the three models are robust.

### 4.4. Influence Mechanism of the Happiness for Land-Expropriated, Urbanised Residents

The group (Sample D) who attained urban hukou through education and job recruitment had the highest level of happiness. In this section, Sample D was taken as the reference group in an attempt to explore the influence mechanism of the loss of happiness of the land-expropriated, urbanised residents (Sample C). Here, Samples C and D were combined into research samples, and 0–1 dummy variables of the conversion path were established. When the ’nonagriculture' hukou status was achieved through land acquisition, it was assigned a value of 1; conversely, when the process was achieved by selective mobility, such as education and job recruitment, it was assigned a value of 0. In the context of urbanisation, with the increase in land value and government economic compensation, the farmers who obtained urban hukou through land acquisition were full of expectations for a better life after entering the city. Therefore, the expectation of future life may be the intermediary that affected the happiness of the group. On the basis of the data from the CGSS in 2017 and 2018, this study took ‘subjective class score 10 years from now’ minus the ‘subjective class score now’ as a measure of future living expectation. The higher the score was, the more hopeful they would be about their future life. Conversely, the future life was full of pessimism. Happiness is not only about future expectations but also about current life situations. The differences in the pathway of hukou transition may lead to different self-evaluations, including self-assessment of socioeconomic status. The CGSS asked the following question: ‘What is your socioeconomic status in the current society?’ According to the answers to this question, an individual’s socioeconomic status was set to 1–5, with a higher value indicating higher status. Similarly, this study hypothesised that the self-assessment socioeconomic status played a mediating effect. Table 6 and Table 7 show the corresponding estimation results.

Model 4 in Table 6 shows that the pathway of hukou transition had a significantly negative effect on happiness, that is, the land-expropriated, urbanised residents were significantly lower than the residents who attained urban hukou through education and job recruitment. According to the procedure of the mediating effect, the second step is to examine whether a significant effect of the pathway of hukou transition exists on living expectancy. The regression coefficient of Model 5 was 0.1156, which was significant at the 0.1 level. Thus, the land-expropriated, urbanised residents had a higher living expectation. When the pathway of hukou transition and living expectation entered into Model 6 simultaneously, the regression coefficient of the former was −0.1018 and was significant at the level of 0.05. The latter was 0.0599 and was significant at the level of 0.01. In this case, the direct effect of the pathway of hukou transition on happiness was significant, and the indirect effect of living expectation was also significant. However, according to the idea of Mackinnon et al. (2000) [27], the indirect effect is not the mediating effect but the suppressing effect. The indirect effect was opposite to the direct effect, and the absolute value of the total effect (−0.0948) in Model 4 was smaller than that of the direct effect (−0.1018). Hence, land acquisition increased the level of happiness by raising the expectation of future life, but it concealed the negative effect on happiness.

Self-assessment of socioeconomic status is an individual’s subjective evaluation of the current life status. The regression results are shown in Table 7. The results of Model 7 are similar to those of Model 4 in Table 6, which indicate that the land-expropriated, urbanised residents had a lower happiness level. In Model 8, the coefficient of the pathway of hukou transition was −0.1745 and was significant at the 0.01 level. In comparison with those who achieve urban hukou through education and job recruitment, the land-expropriated, urbanised residents had a lower evaluation of their social and economic status. In the third step of Model 9, the regression coefficients of self-assessment of socioeconomic status and the pathway of hukou transition were significant at the 0.01 and 0.1 levels, respectively. At the same time, the indirect effect had the same sign as the direct effect. Therefore, the indirect effect was a partial mediating effect. In the process of hukou transition through land expropriation, there would be loss of happiness, part of which would be caused by the subjective evaluation of the self-assessment of socioeconomic status.

## 5. Conclusions and Inspiration

On the basis of the data from the CGSS in 2017 and 2018, the samples were divided into farmers (9274), urban native residents (6704), land-expropriated, urbanised residents (614) and residents who attained urban hukou through education and job recruitment (1513). The HeckProbit model, ordered probit model and mediating effect model were adopted to study the happiness and underlying mechanism of the land-expropriated, urbanised residents.

The results of the HeckProbit model showed that the older the residents were, the lower the probability of hukou conversion through land acquisition, and women would be more likely to achieve household registration through land acquisition. The higher the individual education was, the lower the probability of achieving the hukou transition through land acquisition. Party status had a significant inhibitory effect on the hukou conversion through land acquisition.

The results of the ordered probit model showed that the residents who attained urban hukou through education and job recruitment had the highest level of happiness, followed by the urban native residents, whereas no significant difference existed in the happiness between the land-expropriated, urbanised residents and the farmers. The propensity score matching method was further used to reduce the endogenous problem, and the result remained robust.

Finally, the mediating effect model demonstrated that living expectation and self-assessment of socioeconomic status played suppressing and mediating effects between the pathway of hukou transition and happiness, respectively.

On the basis of the above conclusions, the following policy suggestions are proposed. First, policy emphasis should be placed on urbanisation centred on improving the happiness of residents. In the past, urbanisation mostly emphasised the urbanisation of land and population, ignoring that the essence of urbanisation is to enhance the happiness of residents. For future urbanisation and land acquisition system reform, this study suggests introducing an evaluation indicator for residents’ happiness as a measure of government performance. Second, this study suggests strengthening policy interpretations and publicity and avoiding realising rural-to-urban hukou conversion through land acquisition blindly. Although the government has given some economic compensation, the human capital stock of the group is generally low. Certain livelihood risks exist for farmers who acquire urban hukou blindly. Therefore, the government should objectively publicise the policies related to land acquisition, such that farmers can make rational choices based on understanding the policies and combining their own endowments. Third, efforts should be made to further improve the comprehensive security measures for land-expropriated farmers. Governments at all levels need to further improve the medical and social security levels of this group and protect their legal rights and interests. In particular, employment skill training should be intensified to lay a foundation for this group to ‘root’ in the city and lead a happy life.

## Figures and Tables

**Table 1:** Descriptive statistics.

Categorical Variables	Sample A	Sample B	Sample C	Sample D
Gender				
male	4287 (46.23%)	3233 (48.22%)	286 (46.58 %)	854 (56.44%)
female	4987 (53.77%)	3471 (51.78%)	328 (53.42%)	659 (43.56%)
Education				
without formal education	2291 (24.70%)	263 (3.92 %)	92 (14.98 %)	63 (4.16%)
elementary school	3231 (34.84%)	466 (6.95 %)	159 (25.90%)	134 (8.86 %)
junior high school	2657 (28.65%)	1539 (22.96%)	215 (35.02%)	224 (14.81%)
high school	830 (8.95%)	1901 (28.36 %)	93 (15.15%)	344 (22.74 %)
junior college and above	265 (2.86%)	2535 (37.81 %)	55 (8.96 %)	748 (49.44 %)
Party status				
party member	674 (7.27%)	1254 (18.71%)	55 (8.96 %)	479 (31.66%)
others	8600 (92.73%)	5450 (81.29%)	559 (91.04 %)	1034 (68.34 %)
Parents’ Education				
without formal education	5718 (61.66%)	1864 (27.80%)	335 (54.56%)	519 (34.30 %)
elementary school	1898 (20.47%)	1387 (20.69%)	149 (24.27 %)	405 (26.77%)
junior high school	755 (8.14%)	1142 (17.03 %)	62 (10.10%)	261 (17.25%)
high school	279 (3.01%)	1076 (16.05%)	32 (5.21 %)	185 (12.23%)
junior college and above	624 (6.73%)	1235 (18.42%)	36 (5.86 %)	143 (9.45%)
Parents’ party status				
party member	584 (93.56 %)	1316 (20.21%)	58 (9.60%)	249 (16.84%)
others	8488 (6.44 %)	5197 (79.79 %)	546 (90.40 %)	1230 (83.16%)
Marital cohabitation				
unmarried, separated but not divorced, divorced and widowed	1759 (18.97%)	1824 (28.01 %)	108 (17.88 %)	222 (15.01%)
others	7515 (81.03%)	4689 (71.99%)	496 (82.12%)	1257 (84.99%)
Health status				
very unhealthy	632 (6.82%)	128 (1.98%)	22 (3.67%)	35 (2.39%)
relatively unhealthy	2122 (22.89%)	663 (10.27%)	100 (16.69%)	167 (11.42%)
general	2233 (24.08%)	1729 (26.79%)	135 (22.54%)	356 (24.35%)
relatively healthy	2944 (31.75%)	2660 (41.21%)	21,435.73 (%)	610 (41.72%)
very healthy	1341 (14.46%)	1274 (19.74%)	128 (21.37%)	294 (20.11%)
Family economic status				
far below average	1049 (11.35%)	212 (3.28%)	48 (8.01%)	39 (2.67%)
below average	3779 (40.88%)	1824 (28.25%)	220 (36.73%)	378 (25.84%)
average	4062 (43.94%)	3688 (57.12%)	296 (49.42%)	855 (58.44%)
above average	335 (3.62%)	707 (10.95%)	33 (5.51%)	188 (12.85%)
far above average	20 (0.22%)	26 (0.40%)	20.33 (%)	3 (0.21%)
Self-assessment of socioeconomic status				
upper level	2842 (30.64%)	795 (12.26%)	120 (20.24%)	163 (11.08%)
middle upper level	3207 (34.58%)	2215 (34.16%)	218 (36.76%)	504 (34.26%)
middle level	2895 (31.22%)	2924 (45.10%)	22,137.27 (%)	675 (45.89%)
middle lower level	305 (3.29%)	533 (8.22%)	34 (5.73%)	126 (8.57%)
lower level	25 (0.27%)	17 (0.26%)	0	3 (0.20%)
Happiness				
very unhappy	180 (1.94%)	46 (0.71%)	6 (1.01%)	12 (0.82%)
relatively unhappy	824 (8.89%)	154 (2.38%)	416.91 (%)	46 (3.13%)
not happy or unhappy	1357 (14.63%)	783 (12.08%)	72 (12.14%)	132 (8.97%)
relatively happy	5358 (57.77%)	4137 (63.8%)	348 (58.68%)	933 (63.43%)
very happy	1555 (16.77%)	1364 (21.04%)	12,621.25 (%)	348 (23.66%)
**Numeric variables**				
Age	55.34408 (14.8065)	51.5108 (17.6874)	54.2345 (14.8013)	52.6483 (16.8955)
Number of real estates	0.08343 (0.2455)	0.1668 (0.3410)	0.2220 (0.4279)	0.1872 (0.3607)
Living expectation	0.6837 (1.2443)	0.6020 (1.1802)	0.7723 (1.3756)	0.6505 (1.2460)

Note: mean and standard deviations in brackets for numeric variables, sample size and proportion in brackets for categorical variables.

**Table 2:** HeckProbit model estimation.

Variables	Outcome Equation	Selection Equation
Age	−0.0171 ***	0.0184 ***
	(0.00371)	(0.00123)
Gender	−0.0351	−0.146 ***
	(0.0658)	(0.0320)
Education	−0.128 ***	0.171 ***
	(0.0303)	(0.00499)
Party status	−0.327 ***	0.499 ***
	(0.103)	(0.0487)
Parents’ education		0.0278 ***
		(0.00388)
Parents’ party status		0.164 ***
		(0.0510)
Constants	1.330 **	−3.455 ***
	(0.639)	(0.0978)
*rho*	0.369 **	
	(0.154)	
LR test statistics	3.98 **	
Sample size	11,155	

Note: (1) standard error in brackets; (2) ***, ** indicate significant at 1%, 5% levels, respectively.

**Table 3:** Comparison of happiness among multiple groups.

Variables	Happiness		
	Model 1	Model 2	Model 3
Household register	0.0112	−0.0433 *	0.0232 **
	(0.0485)	(0.0262)	(0.0117)
Age	0.0119 ***	0.00659 ***	0.00608 ***
	(0.000934)	(0.000979)	(0.000916)
Gender	−0.112 ***	−0.142 ***	−0.175 ***
	(0.0238)	(0.0288)	(0.0273)
Marital cohabitation	0.0922 ***	0.147 ***	0.158 ***
	(0.0298)	(0.0334)	(0.0322)
Education	0.0182 ***	0.00345	0.00196
	(0.00361)	(0.00467)	(0.00443)
Health status	0.209 ***	0.0783 ***	0.0545 ***
	(0.0112)	(0.00886)	(0.00678)
Family economic status	0.342 ***	0.385 ***	0.363 ***
	(0.0166)	(0.0216)	(0.0205)
Number of real estates	0.285 ***	0.133 ***	0.125 ***
	(0.0453)	(0.0422)	(0.0406)
Sample size	9368	6393	7161
Pseudo R^2^	0.0559	0.0445	0.0394

Note: (1) Standard errors are shown in brackets; (2) ***, ** and * indicate significance at the 1%, 5% and 10% levels, respectively. Due to space limitations, the tangent point estimates are omitted here.

**Table 4:** Sample balance test.

Variables	Treatment Group	Control Group	Standard Deviation (%)	Deviation Reduction (%)	T Value	*p* Value
Age						
Before matching	54.103	55.335	−8.4		−1.96	0.050
After matching	54.103	54.406	−2.1	75.4	−0.35	0.729
Gender						
Before matching	0.46998	0.46361	1.3		0.30	0.765
After matching	0.46998	0.46029	1.9	−52.1	0.33	0.740
Marital cohabitation						
Before matching	0.82333	0.81431	2.3		0.54	0.587
After matching	0.82333	0.82617	−0.7	68.4	−0.13	0.898
Education						
Before matching	8.0429	6.4302	41.9		10.05	0.000
After matching	8.0429	8.1202	−2.0	95.2	−0.34	0.737
Health status						
Before matching	3.5437	3.2664	18.3		3.60	0.000
After matching	3.5437	3.3975	9.7	47.3	2.23	0.026
Family economic status						
Before matching	3.3568	2.7169	8.7		2.63	0.009
After matching	3.3568	3.4262	−0.9	89.2	−0.13	0.896
Number of real estates						
Before matching	0.22091	0.09768	31.5		8.07	0.000
After matching	0.22091	0.177	11.2	64.4	1.65	0.099

**Table 5:** Results based on propensity score matching.

Sample	Treatment Group	Control Group	Difference
Before matching	3.9091	3.7901	0.1190
ATT	3.9091	3.8756	0.0335
ATU	3.7906	3.8225	0.0320
ATE			0.0321

**Table 6:** Estimation of living expectation mechanism.

	Model 4	Model 5	Model 6
Variables	Dependent variable: Happiness	Dependent variable: Living expectation	Dependent variable: Happiness
Living expectation			0.0599 ***
			(0.0143)
Pathway of hukou transition	−0.0948 **	0.1156 *	−0.1018 **
	(0.0423)	(0.0675)	(0.0421)
Control variables	Controlled	Controlled	Controlled

Note: ***, ** and * indicate significance at the 1%, 5% and 10% levels, respectively.

**Table 7:** Estimation of socioeconomic status mechanism.

	Model 7	Model 8	Model 9
Variables	Dependent variable: Happiness	Dependent variable: Self-assessment of socioeconomic status	Dependent variable: Happiness
Self-assessment of socioeconomic status			0.2201 ***
			(0.0205)
Pathway of hukou transition	−0.1066 **	−0.1745 ***	−0.0682 *
	(0.0421)	(0.0452)	(0.0410)
Control variables	Controlled	Controlled	Controlled

Note: ***, ** and * indicate significance at the 1%, 5% and 10% levels, respectively.

## Data Availability

The data presented in this study are available on request from the corresponding author.
